# Prognostic value of a nomogram based on peripheral blood immune parameters in unresectable hepatocellular carcinoma after intensity-modulated radiotherapy

**DOI:** 10.1186/s12876-022-02596-0

**Published:** 2022-12-09

**Authors:** Jian-Xu Li, Mei-Ling He, Mo-Qin Qiu, Liu-Ying Yan, Mei-Ying Long, Jian-Hong Zhong, Rui-Jun Zhang, Chun-Feng Liang, Ya-Dan Pang, Jun-Kun He, Qian-Qian Chen, Jin-Xia Weng, Shi-Xiong Liang, Bang-De Xiang

**Affiliations:** 1 Department of Radiation Oncology, Guangxi Medical University Cancer Hospital, Nanning, 530021 China; 2 Department of Respiratory Oncology, Guangxi Medical University Cancer Hospital, Nanning, 530021 China; 3 Department of General Affairs, Guangxi Medical University Cancer Hospital, Nanning, 530021 China; 4 School of Public Health, Guangxi Medical University, Nanning, 530021 China; 5 Department of Hepatobiliary Surgery, Guangxi Medical University Cancer Hospital, Nanning, 530021 China

**Keywords:** Hepatocellular carcinoma, Immune parameters, Nomogram, Intensity-modulated radiotherapy

## Abstract

**Background:**

For patients with unresectable hepatocellular carcinoma (uHCC), intensity-modulated radiotherapy (IMRT) has become one of the options for clinical local treatment. Immune parameters, including platelet-to-lymphocyte ratio (PLR), neutrophil-to-lymphocyte ratio (NLR) and systemic immune inflammatory (SII), predict survival in various cancers. This study aimed to determine whether peripheral immune parameters can predict survival in patients with uHCC undergoing IMRT and establish a clinically useful prognostic nomogram for survival prediction.

**Methods:**

The clinical data of 309 HCC patients were retrospectively analyzed and randomly divided into training (n = 216) and validation (n = 93) cohorts. PLR, NLR and SII were collected before and after IMRT. Univariate and multivariate Cox analyses were performed to identify independent prognostic factors affecting survival, which were used to generate a nomogram.

**Results:**

The median survival was 16.3 months, and significant increases in PLR, NLR, and SII were observed after IMRT (*P* < 0.001). High levels of immune parameters were associated with poor prognosis (*P* < 0.001); enlarged spleen, Barcelona clinic liver cancer stage (B and C), post-SII, and delta-NLR were independent risk factors for survival and were included in the nomogram, which accurately predicted 3- and 5-year survival. The nomogram was well verified in the validation cohort.

**Conclusions:**

High levels of immune parameters are associated with poor prognosis in uHCC patients receiving IMRT. Our nomogram accurately predicts the survival of patients with uHCC receiving IMRT.

**Supplementary Information:**

The online version contains supplementary material available at 10.1186/s12876-022-02596-0.

## Introduction

Hepatocellular carcinoma (HCC) is the third major cause of cancer-related deaths worldwide. Approximately 70% of HCC patients have unresectable disease at the time of diagnosis [1]. For patients with unresectable HCC (uHCC), the recommended multikinase inhibitors showed disappointing results. However, the immune checkpoint inhibitors or combination with biological therapy are new promising treatment options [2]. But the treatment outcomes in patients with uHCC remain unsatisfactory. Radiotherapy (RT) has been recommended as a standard clinical local treatment for uHCC [3].

The clinical efficacy of RT is mainly attributed to the direct effect of ionizing radiation on double-stranded DNA, which directly leads to tumor cell death [4]. With the development of tumor immunotherapy, RT has been found to trigger complex immune responses and affect the tumor microenvironment and the system immune cells [5, 6]. Peripheral immune parameters are considered an important part of the tumor microenvironment, and tumor-related inflammation plays an important role in tumor occurrence and development and is also believed to lead to tumor cell invasion and metastasis [7]. Studies have identified and validated the abundance and ratio of peripheral immune cells, such as high neutrophil-to-lymphocyte ratio (NLR), platelet-to-lymphocyte ratio (PLR) and lymphocyte-to-monocyte ratio (LMR) as novel prognostic biomarkers in various cancers [8–10]. For HCC, high NLR and PLR predicted lower survival and a higher risk of early recurrence after resection, sorafenib, TACE and liver transplantation [11–14]. Systemic immune-inflammation index (SII) can reflect the balance between host immunity and immune status of HCC [15].

Therefore, we retrospectively evaluated the peripheral immune parameters before and after intensity-modulated radiotherapy (IMRT) and their prognostic value in predicting the survival outcome of uHCC patients. We aimed to establish a prognostic model to accurately predict the survival in patients with uHCC receiving IMRT.

## Methods

### Patient characteristics

A retrospective study was performed on 309 HCC patients who received IMRT treatment at Guangxi Medical University Cancer Hospital from February 2013 to July 2021. Patients with uHCC were included as per the following criteria: (a) diagnosed pathologically or according to the criteria of the European Association for the Study of the Liver, (b) Child–Pugh grade A or B liver function, and (c) completed peripheral blood cell counts before and after IMRT. Patients who underwent postoperative adjuvant IMRT, failed follow-up, and were unable to have their peripheral blood cell count monitored were excluded. This study was approved by the institutional review board of Guangxi Medical University Cancer Hospital (number LW2022059).

### IMRT

All patients underwent enhanced computerized tomography (CT) scan at 2.5–5 mm slice thickness for the IMRT plan. Gross tumor volume (GTV) was defined as tumor focus. CT-positron emission tomography fusion and magnetic resonance imaging fusion for extrahepatic metastasis and intrahepatic lesions were performed, respectively. The GTV and organs at risk were contoured on the Pinnacle 3 system (Philips, Netherlands) or MIM software (version 6.8; MIM, USA). The planned target volume (PTV) was defined as GTV plus asymmetrical dilation of 1 cm in the craniocaudal direction and 5 mm in the axial direction to set uncertainty and respiratory movement. The IMRT plans were designed using the Pinnacle 3 system or Monaco treatment planning system version 5.1. The final median biologically effective dose, which used α/β ratio = 10 according to the linear-quadratic model, was 67.2 Gy (interquartile range, 60–78 Gy). IMRT was delivered via a 6 MV X-ray linear accelerator (ELEKTA Synergy or ELEKTA Versa-HD, Sweden) using cone-beam CT to correct the positions.

### Peripheral blood immune parameters

Peripheral blood was collected within 1 week before IMRT to determine lymphocyte, neutrophil, and platelet counts. The ratios and changes in values were calculated as follows: PLR = platelet count/lymphocyte count; NLR = neutrophil count/lymphocyte count; SII = platelet count × neutrophil count/lymphocyte count; post-PLR = post-platelet count/post-lymphocyte count; post-NLR = post-neutrophil count/post-lymphocyte count; post-SII = post-platelet count × post-neutrophil count/post-lymphocyte count; delta-NLR = NLR − post-NLR; delta-PLR = PLR − post-PLR; and delta-SII = SII − post-SII. The optimal cutoff value of immune parameters was selected according to the area under the receiver operating characteristic (ROC) curve.

### Statistical analysis

R language (version 4.0.3) was used for statistical analysis. To validate model performance, 309 uHCC patients were randomly divided into the training and validation cohorts. The differences in baseline characteristics of the patients in these two cohorts were examined by χ^2^ tests and Student’s *t*-tests for categorical and continuous variables, respectively. The overall survival (OS) was defined as the day of informed consent to the day of death from any cause. The survival curves were drawn using the Kaplan–Meier method, and the difference was compared using the log-rank test. The Cox proportional hazard model was used to determine the prognostic indicators, and the significant variables (*P* < 0.05) in the univariate Cox model were included in the multivariate model. Backward stepwise selection (*P* = 0.05 for removal) was used to select characteristics for the multivariable regression model. The nomogram was constructed based on the variables included in the final multivariate Cox proportional hazard model. Moreover, the efficiency of the nomogram was evaluated, and the time-dependent ROC curve and area under the ROC curve (AUROC) were analyzed to compare the discriminatory ability of different models in total survival. Statistical significance was set at *P* < 0.05.

## Results

### Clinicopathological features of patients

**Fig. 1:**
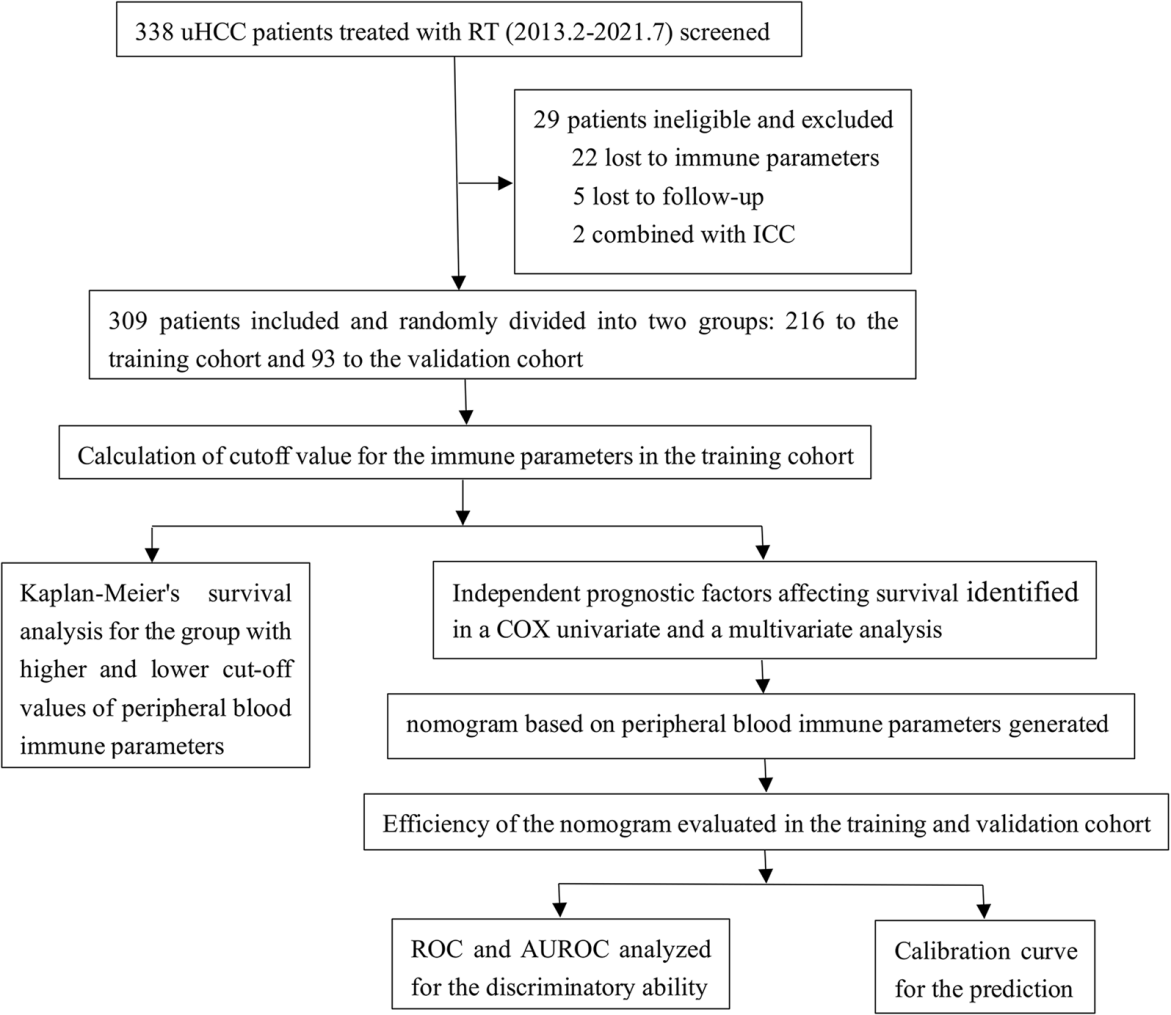
Study flow. *uHCC* unresectable hepatocellular carcinoma, *RT* radiotherapy, *ICC* intrahepatic cholangiocarcinoma

A total of 338 patients with uHCC were screened, and 309 were included in the study (Fig. 1). The participants included 280 males and 29 females, with a mean age of 55 years, median OS of 16.3 months, and a median follow-up of 38.8 months. Most patients had Child–Pugh grade A liver function (238 cases; 77%), Barcelona clinic liver cancer (BCLC) stage C (250 cases; 80.9%), and radiographic cirrhosis (212 cases; 68.6%). There were 175 cases of splenomegaly. Before IMRT, transcatheter arterial chemoembolization (TACE) (66%) and resection (48.2%) were the main treatment methods. After IMRT, TACE (17.5%), targeted therapy (12%), and immunization (11.7%) were the most common treatment methods. All clinicopathological parameters are shown in Table 1. The patients were randomly divided into the training cohort (n = 216) and validation cohort (n = 93), and no significant difference in baseline characteristics was observed between the two cohorts (*P* > 0.05).

**Table 1:** Baseline characteristics of the patients in the training cohort and validation cohort

Variables	Total, n = 309 (%)	Training cohort, n = 216(%)	Validation cohort, n = 93(%)	*P* value
*Age*				
≦ 60 years	210 (68.0)	153 (70.8)	57 (61.3)	0.13
> 60 years	99 (32.0)	63 (29.2)	36 (38.7)	
*Sex*				
Female	29 (9.4)	23 (10.6)	6 (6.5)	0.343
Male	280 (90.6)	193 (89.4)	87 (93.5)	
*Liver cirrhosis*				
No	97 (31.4)	65 (30.1)	32 (34.4)	0.538
Yes	212 (68.6)	151 (69.9)	61 (65.6)	
*ECOG PS*				
0	63 (20.4)	46 (21.3)	17 (18.3)	0.414
1	240 (77.7)	167 (77.3)	73 (78.4)	
2	5 (1.6)	3 (1.4)	2 (2.2)	
3	1 (0.3)	0 (0.0)	1(1.1)	
*Pre-RT AFP*				
< 400 ng/ml	195 (63.1)	137 (63.4)	58 (62.4)	0.961
≧ 400 ng/ml	114 (36.9)	79 (36.6)	35 (37.6)	
*HBV infection*				
No	58 (18.8)	38 (17.6)	20 (21.5)	0.516
Yes	251 (81.2)	178 (82.4)	73 (78.5)	
TBIL (mean (SD))	17.59 (12.96)	17.10 (12.50)	18.72 (13.88)	0.315
Alb (mean (SD))	35.43 (4.57)	35.63 (4.55)	35.94 (4.58)	0.232
*Enlarged spleen*				
No	134 (43.4)	92 (42.6)	42 (45.2)	0.77
Yes	175 (56.6)	124 (57.4)	51 (54.8)	
*Largest tumor size*				
≦ 6 cm	155 (50.2)	115 (53.2)	40 (43.0)	0.127
> 6 cm	154 (49.8)	101 (46.8)	53 (57.0)	
*Number of tumors*				
≦ 3	154 (49.8)	116 (53.7)	38 (40.9)	0.052
> 3	155 (50.2)	100 (46.3)	55 (59.1)	
*Macrovascular invasion*				
No	130 (42.1)	92 (42.6)	38 (40.9)	0.875
Yes	179 (57.9)	124 (57.4)	55 (59.1)	
*Extrahepatic metastases*				
No	183 (59.2)	128 (59.3)	55 (59.1)	1
Yes	126 (40.8)	88 (40.7)	38 (40.9)	
*BCLC stage*				
A	26 (8.4)	20 (9.3)	6 (6.4)	0.644
B	33 (10.7)	24 (11.1)	9 (9.7)	
C	250 (80.9)	172 (79.6)	78 (83.9)	
*Child–Pugh class*				
A	238 (77.0)	167 (77.3)	71 (76.3)	0.969
B	71 (23.0)	49 (22.7)	22 (23.7)	
*Prior treatment*				
TACE	204 (66.0)	143 (66.2)	61 (65.6)	1
RFA	57 (18.4)	41 (19.0)	16 (17.2)	0.834
Surgery	149 (48.2)	110 (50.9)	39 (41.9)	0.185
Targeted therapy	25 (8.1)	19 (8.8)	6 (6.5)	0.641
Immunotherapy	12 (3.9)	9 (4.2)	3 (3.2)	0.943
*Concurrent treatment*				
Targeted therapy	4 (1.3)	3 (1.4)	1 (1.1)	1
Immunotherapy	28 (9.1)	18 (8.3)	10 (10.8)	0.643

Data are mean ± standard deviation or N (%)

*RT* Radiotherapy, *TBIL* Total bilirubin, *Alb* Albumin, *AFP* Alpha-fetoprotein, *HBV* Hepatitis B virus, *BCLC* Barcelona clinic liver cancer, *TACE* Transcatheter arterial chemoembolization, *RFA* Radiofrequency ablation

### Changes in immune parameters before and after IMRT

Peripheral blood was collected 1 week before and 1 month after IMRT. The immune parameters of all patients are shown in Additional file 1: Table S1. Statistical analysis of peripheral blood and inflammatory indicators showed that neutrophil and platelet counts decreased significantly after IMRT (both *P* < 0.001), while no significant difference was observed in lymphocyte counts. Similarly, a significant increase in inflammatory indicators (PLR, NLR, SII) was observed after IMRT (all *P* < 0.001).

### Immune parameters and survival outcomes

**Fig. 2:**
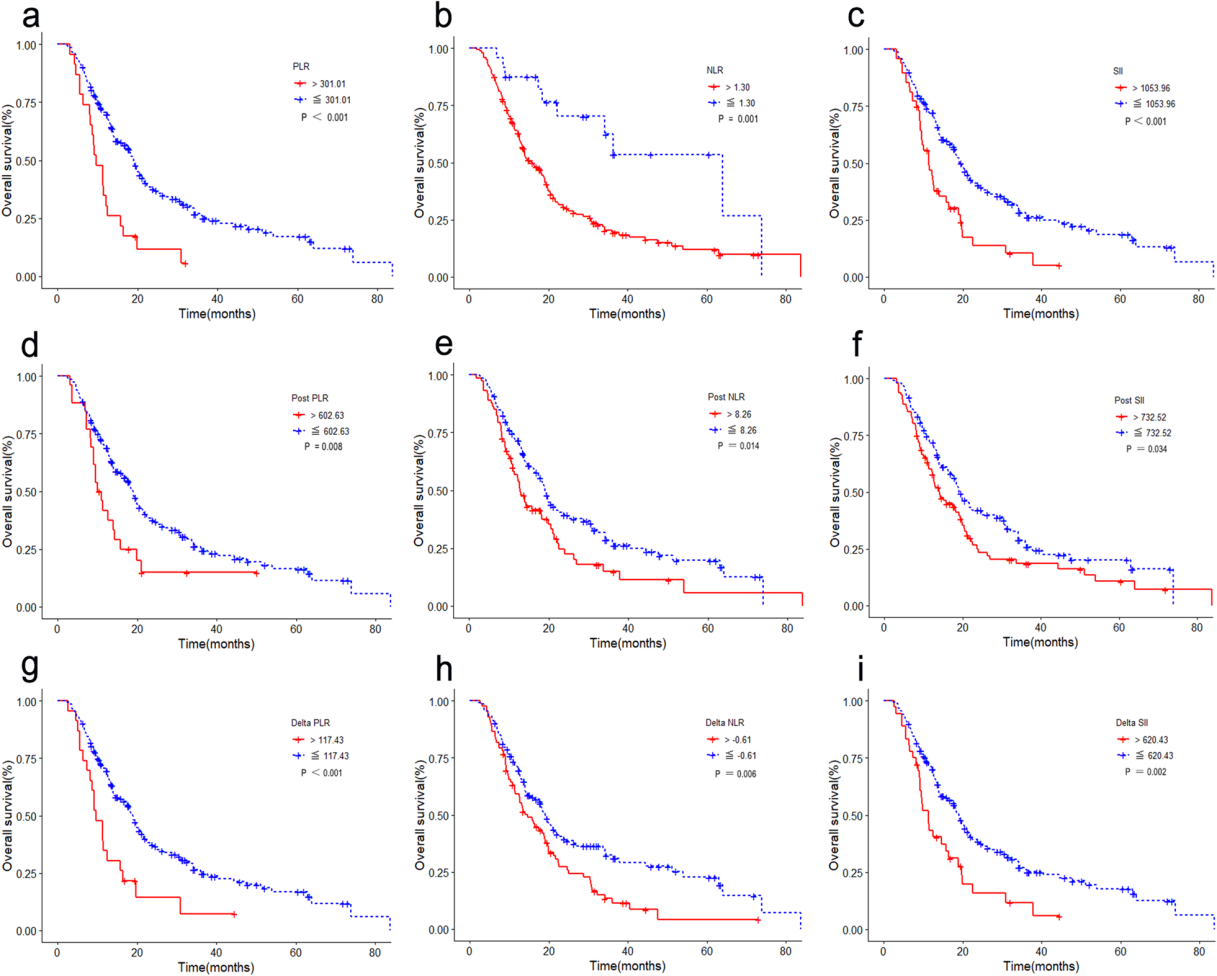
The prognostic significance of the Immune parameters in the training cohort. Kaplan–Meier survival analysis showed the HCC prognosis in the group with the cutoff value of PLR (**a**), NLR (**b**), SII (**c**), post-PLR (**d**), post-NLR (**e**), post-SII (**f**), delta-PLR (**g**), delta-NLR (**h**), and delta-SII (**i**). PLR = platelet count/lymphocyte count; NLR = neutrophil count/lymphocyte count; SII = platelet count × neutrophil count/lymphocyte count. Post PLR = post-platelet count/post-lymphocyte count; post NLR = neutrophil count/ post-lymphocyte count; post SII = post-platelet count × post-neutrophil count/post-lymphocyte count; delta NLR = NLR − post NLR; delta PLR = PLR − post PLR; delta SII = SII − post SII

In the validation cohort, We divided the immune parameters into high and low groups according to the cutoff values and found that all of them affected the prognosis of patients with uHCC after IMRT: PLR ≤ 301.01 and PLR > 301.01 (hazard ratio [HR] 2.48, 95% confidence interval [95% CI] 1.55–3.97, *P* = 0.000), NLR ≤ 1.3 and NLR > 1.3 (HR 2.79, 95% CI 1.47–5.32, *P* = 0.002), SII ≤ 1053.96 and SII > 1053.96 (HR 2.04, 95% CI 1.40–2.95, *P* = 0.000), post-PLR ≤ 602.63 and post-PLR > 602.63 (HR 1.87, 95% CI 1.17–2.97, *P* = 0.009), post-NLR ≤ 8.26 and post-NLR > 8.26 (HR 1.51, 95% CI 1.08–2.11, *P* = 0.015), post-SII ≤ 732.52 and post-SII > 732.52 (HR 1.41, 95% CI 1.03–1.93, *P* = 0.034), delta-PLR ≤ 117.43 and delta-PLR > 117.43 (HR 2.18, 95% CI 1.36–3.51, *P* = 0.001), delta-NLR ≤ − 0.61 and delta-NLR > -0.61 (HR 1.57, 95% CI 1.14–2.16, *P* = 0.006), delta-SII ≤ 620.43 and delta-SII > 620.43 (HR 1.88, 95% CI 1.26–2.81, *P* = 0.002). Kaplan–Meier survival analysis showed a worse HCC prognosis in the group with immune parameters values higher than the cutoff value (Fig. 2a–i). In contrast, neutrophil, lymphocyte, and platelet counts had no significant effect on prognosis. Cox univariate analysis revealed that enlarged spleen, tumor number, tumor size, distant metastasis, BCLC stage (B, C), surgical treatment before IMRT, total bilirubin level, lymphocyte count, NLR, and SII were all prognostic factors, while Sex, age, liver cirrhosis, HBV infection, vascular invasion, TACE before IMRT, RFA treatment and synchronization, and follow-up treatment had no significant effect on prognosis (Table 2). Multivariate analysis further showed that enlarged spleen, BCLC stage (B and C), post-SII, and delta-NLR were independent risk factors for uHCC (Table 2).

**Table 2:** Univariate and multivariate Cox regression analyses for overall survival

Characteristics	Univariable analysis		Multivariable analysis	
	HR (95%CI)	*P* value	HR (95%CI)	*P* value
Sex (male vs female)	1.35 (0.79–2.3)	0.280		
Age year (> 60 vs ≦ 60)	0.99 (0.98–1.01)	0.478		
Liver cirrhosis (yes vs no)	1.01 (0.72–1.43)	0.937		
ECOG PS	1.1 (0.74–1.63)	0.636		
AFP ng/ml (≧ 400 vs < 400)	1.34 (0.97–1.86)	0.078		
HBV infection (yes vs no)	0.93 (0.61–1.43)	0.755		
Enlarged spleen (yes vs no)	1.54 (1.11–2.13)	0.010	1.45 (1.00, 2.10)	0.047
MVI (yes vs no)	1.1(0.8–1.52)	0.549		
Largest tumor size cm (> 6 vs ≦ 6)	1.61 (1.17–2.22)	0.003	0.87 (0.56, 1.34)	0.523
Number of tumors (> 3 vs ≦ 3)	1.82 (1.32–2.5)	0.000	1.38 (0.96, 2.00)	0.084
Extrahepatic metastases (yes vs no)	1.39 (1.01–1.91)	0.044	1.37 (0.93, 2.03)	0.111
BCLC stage				
B versus A	4.03 (1.71–9.51)	0.001	3.89 (1.50, 10.11)	0.005
C versus A	3.75 (1.75–8.04)	0.001	2.59 (1.07, 6.24)	0.034
Child–Pugh class (B vs A)	1.42 (0.98–2.06)	0.064		
Prior treatment				
TACE (yes vs no)	1.02(0.73–1.42)	0.893		
RFA (yes vs no)	0.84 (0.55–1.28)	0.407		
Surgery (yes vs no)	0.64 (0.47–0.88)	0.006	0.82 (0.54, 1.22)	0.324
Targeted therapy (yes vs no)	1.29 (0.69–2.4)	0.424		
Immunotherapy (yes vs no)	1.48 (0.6–3.62)	0.396		
Concurrent treatment				
Targeted therapy (yes vs no)	0.36 (0.05–2.58)	0.309		
Immunotherapy (yes vs no)	1.04(0.53–2.06)	0.903		
Post treatment				
TACE (yes vs no)	0.81 (0.54–1.21)	0.295		
RFA (yes vs no)	0.77 (0.31–1.88)	0.562		
Surgery (yes vs no)	0.51(0.21–1.26)	0.145		
Targeted therapy (yes vs no)	0.81(0.49–1.33)	0.401		
Immunotherapy (yes vs no)	1.06 (0.63–1.78)	0.835		
Radiation therapy (yes vs no)	0.6 (0.28–1.3)	0.199		
TBIL	1.01 (1–1.02)	0.044	1.01 (1.00, 1.02)	0.122
Alb	0.98 (0.94–1.01)	0.164		
Platelet count	1 (1.00–1.00)	0.408		
Neutrophil count	1.01(0.98–1.04)	0.495		
Lymphocyte count	0.65 (0.49–0.88)	0.005	1.05 (0.72, 1.53)	0.815
Post platelet count	1 (1.00–1.00)	0.694		
Post neutrophil count	0.98 (0.93–1.04)	0.538		
Post lymphocyte count	1.01(0.98–1.03)	0.641		
Delta platelet count	1(1.00–1.00)	0.223		
Delta neutrophil count	1.01 (0.99–1.04)	0.301		
Delta lymphocyte count	0.99 (0.97–1.01)	0.451		
PLR (> 301.01 vs ≤ 301.01)	2.48 (1.55–3.97)	0.000	1.38 (0.63, 3.03)	0.421
NLR (> 1.3 vs ≤ 1.3)	2.79 (1.47–5.32)	0.002	1.24 (0.59, 2.61)	0.570
SII (> 1053.96 vs ≤ 1053.96)	2.04 (1.4–2.95)	0.000	1.60 (0.79, 3.23)	0.190
Post PLR (> 602.63 vs ≤ 602.63)	1.87 (1.17–2.97)	0.009	1.23 (0.70, 2.15)	0.473
Post NLR (> 8.26 vs ≤ 8.26,)	1.51 (1.08–2.11)	0.015	1.31 (0.76, 2.23)	0.330
Post SII (> 732.52 vs ≤ 732.52)	1.41(1.03–1.93)	0.034	2.17 (1.21, 3.88)	0.009
Delta PLR (> 117.43 vs ≤ 117.43)	2.18 (1.36–3.51)	0.001	1.19 (0.52, 2.76)	0.679
Delta NLR (> − 0.61 vs ≤ − 0.61)	1.57 (1.14–2.16)	0.006	2.38 (1.43, 3.95)	0.001
Delta SII (> 620.43 vs ≤ 620.43)	1.88 (1.26–2.81)	0.002	0.82 (0.34, 1.99)	0.665

RT, radiotherapy; TBIL, total bilirubin; Alb, albumin; AFP, alpha-fetoprotein; HBV, hepatitis B virus; MVI, macrovascular invasion; BCLC, Barcelona clinic liver cancer; TACE, transcatheter arterial chemoembolization; RFA, radiofrequency ablation; PLR = platelet count/lymphocyte count; NLR = neutrophil count/lymphocyte count; SII = platelet count × neutrophil count/lymphocyte count; Post-PLR = post-platelet count/post-lymphocyte count; post-NLR = neutrophil count/post-lymphocyte count; post-SII = post-platelet count × post-neutrophil count/post-lymphocyte count; delta-NLR = NLR − post-NLR; delta-PLR = PLR − post-PLR; delta-SII = SII − post-SII

### Establishment and verification of the nomogram

**Fig. 3:**
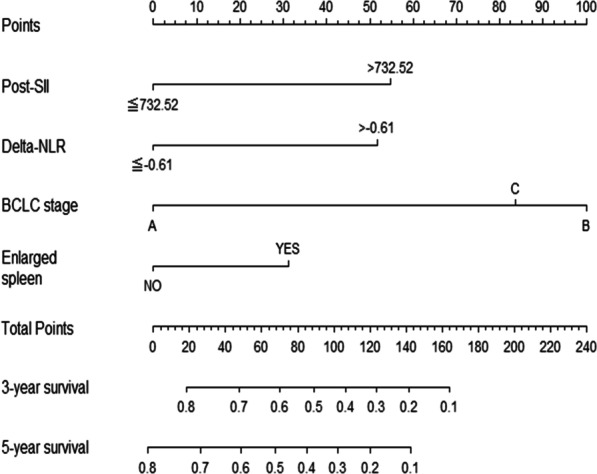
Survival nomogram. The total points of each patient can be used to predict survival outcome. BCLC, Barcelona clinic liver cancer; post SII = post platelet count × post neutrophil count/post-lymphocyte count; delta NLR = NLR − post NLR

Based on the results of multivariate analysis, enlarged spleen, BCLC stage (B, C), post-SII, and delta-NLR were integrated into the nomogram to predict 3- and 5-year survival using the training cohort (Fig. 3). Each category of these variables was assigned a score on the score table, and the overall scores were used to predict survival. Of the variables, the BCLC stage had the most significant effect.

**Fig. 4:**
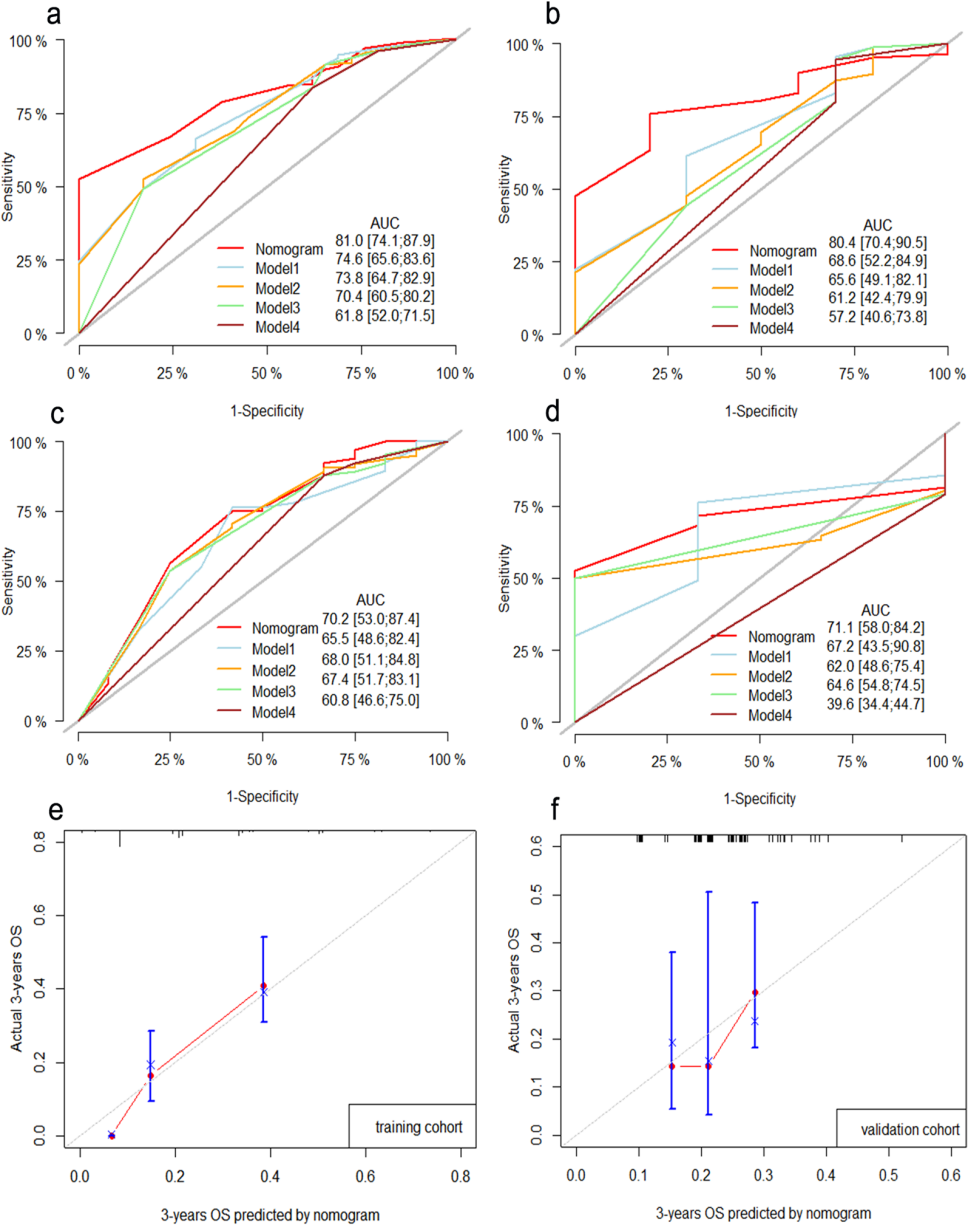
ROC curves and calibration curve for the prediction of survival outcome. ROC curves and AUROCs for the prediction of survival outcome within 3 year (**a**) and 5 years (**b**) among HCC patients after RT in the training cohort, 3 year (**c**) and 5 years (**d**) among HCC patients after RT in the validation cohort. Calibration curve for the prediction of survival outcome within 3 years among uHCC patients after RT in the training cohort (**e**) and validation cohort (**f**). Nomogram: post-SII + delta-NLR + BCLC + enlarged spleen; Model1: delta-NLR + BCLC + enlarged spleen; Model2: post-SII + BCLC + enlarged spleen; Model3: BCLC + enlarged spleen. Model4: BCLC

The 3- and 5-year AUROC values of the training cohort were 81.0 (95% CI 74.1–84.9) and 80.4 (95% CI 70.4–90.5), respectively (Fig. 4a, b), which were higher than those of the other models. Meanwhile, the 3- and 5-year AUROC values of the validation cohort were 70.2 (95% CI 53.0–87.4) and 71.1 (95% CI 58.0–84.2), respectively, which were still higher than those of the other models (Fig. 4c, d).

Moreover, in both cohorts, calibration curves showed that the predicted 3-year survival was consistent with the actual observed results (Fig. 4e, d). Due to the small number of cases followed up for more than 5 years, the calibration curve for 5-year survival could not be drawn. In summary, these results suggest that the nomogram based on enlarged spleen, BCLC stage (B and C), post-SII, and delta-NLR can accurately predict the 3- and 5-year survival rates of HCC patients.

## Discussion

A causal relationship between immune parameters and cancers has become widely accepted. As indicators of immune status, PLR, NLR, and SII have also been studied as prognostic blood biomarkers in the treatment of HCC patients. However, few studies report the association between the immune parameters and survival of patients with uHCC treated with IMRT, and provide an effective model to distinguish patients with worse prognosis. This study provides a new scoring system based on post-SII, delta-NLR, BCLC stage, and enlarged spleen that can effectively evaluate the prognosis of uHCC patients and select suitable patients for IMRT.

In recent studies on HCC patients receiving selective internal RT such as trans-arterial radioembolization recognized as a viable and experimental treatment for intermediate-advanced stage HCC [16], PLR > 290 and post-PLR ≥ 263 were significantly correlated with poor OS and progression-free survival (PFS) [17]. However, Hsiang et al. suggested that neither PLR nor delta-PLR could predict HCC OS or PFS [18]. In our study, PLR (> 301.01), post-PLR (> 602.63), and delta-PLR (> 117.43) were associated with poor OS (*P* < 0.01). In univariate Cox analysis, all of these variables affected OS (*P* < 0.001), although they could not be considered independent factors affecting uHCC OS, they were still used as reference indicators for predicting survival based on their significant correlation with OS.

High NLR indicates low immunity or high inflammation, indicating that the body is in a proinflammatory state and its antitumor activity is weakened, which may suggest a more aggressive disease and poor prognosis [19]. Pre-NLR < 2.1 was correlated with improvement of PFS and OS, and NLR ≥ 2.7 was an independent predictor of HCC OS in advanced HCC patients treated with stereotactic body RT [17, 20]. In our analysis, NLR > 1.3 (*P* = 0.0011) and post-NLR > 8.26 (*P* = 0.014) reflected poor prognosis of HCC. Univariate Cox analysis revealed that NLR > 1.3 (*P* = 0.002) and post-NLR > 8.26, (*P* = 0.015) were factors affecting HCC OS. Delta-NLR ≥ 1.3 was associated with poor OS, and NLR and delta-NLR can complement each other to predict the OS of HCC patients [18]. We analyzed the change in NLR and also found that the delta-NLR > − 0.61 group had poor OS (*P* = 0.0055). Multivariate Cox regression analysis showed that delta-NLR > − 0.61 was an independent prognostic factor for HCC OS (*P* = 0.001). Monitoring of dynamic changes in NLR can provide insight into the balance between immune and inflammatory factors, which can be applied to clinical observation and prediction of the OS of patients with certain guidelines.

SII, a novel prognostic biomarker, can comprehensively reflect the changes in inflammation and peripheral blood counts in the body. In early studies, it was found that increased preoperative SII may be an independent prognostic marker for HCC patients undergoing hepatectomy and liver transplantation and that OS prediction was better than PLR and NLR [21, 22]. In addition, a meta-analysis revealed that high preoperative SII was associated with HCC vascular invasion and tumor diameter, which indirectly suggested poor prognosis [23]. Among studies on HCC patients receiving RT, relatively few have concentrated on SII. The results of this study support the predictive power of SII after IMRT for HCC, and SII > 1053.96, post-SII > 732.52, and delta-SII > 620.43 were all associated with poor OS (*P* < 0.05). Univariate Cox analysis revealed that these three factors affected HCC OS after IMRT, and post-SII was found to be an independent factor of HCC OS in multivariate analysis (*P* = 0.009). SII is a comprehensive indicator of inflammation. Comprehensive analysis of multiple parameters can reduce the influence of changes in a single parameter caused by nontreatment, and it is comprehensive and has been identified as an independent predictor of OS.

After IMRT, immune parameters changed with varying degrees. Survival analysis was performed on the values of and changes in immune parameters before and after IMRT, and the optimal cutoff values were selected for grouping. We found that the values of immune parameters before and after IMRT and the group with values higher than the cutoff were associated with poor prognosis of OS in HCC patients. Markers of immune parameters could be quantified as scores to predict the survival of HCC patients treated with IMRT, while a simple graphical score could be used to estimate survival with a nomogram. Therefore, we determined the independent factors of HCC OS based on multivariate Cox regression analysis and constructed a nomogram to further confirm that the immune parameters affect the prognosis of uHCC patients and may be the predictors of the survival time of uHCC patients after IMRT.

Nomograms have been widely used as a visual tool to predict the survival of HCC patients. Two studies combined NLR with other noninflammatory indicators to construct a nomogram, providing personalized prediction of survival for HCC patients after curative resection [24, 25]. Yu et al. [26] constructed a comprehensive nomogram based on SII and emphasized that SII was an independent prognostic factor affecting the survival outcome of HCC patients after radiofrequency ablation. Based on inflammatory indicators, an HCC nomogram was established, and on the basis of the nomogram, the survival rate of HCC patients at 1, 3, and 5 years was predicted, which was highly accurate and was superior to that of the commonly used American Joint Committee on Cancer TNM staging and BCLC systems after hepatectomy and radiofrequency ablation [27]. However, nomograms are rarely used as a visual tool to predict survival in uHCC patients after IMRT. In our results, post-SII and delta-NLR were found to be independent factors of HCC OS. Based on these two indicators and other commonly used prognostic factors, we constructed a comprehensive nomogram to predict the survival rate of uHCC patients receiving IMRT. The nomogram included the following parameters: enlarged spleen, BCLC stage, post-SII, and delta-NLR. It predicted the 3- and 5-year survival rates, and the correction curve confirmed that the nomogram performed well in predicting 3-year OS of HCC patients and had ideal models in training and validation cohorts, suggesting that the nomogram can well predict OS of uHCC patients in post-SII and delta-NLR performance evaluation.

The commonly used BCLC scoring system for HCC patients assesses prognosis, including liver function grade, tumor load, vascular invasion, and clinical status, but not inflammatory indicators. In addition, patients with BCLC at the same stage may show significantly different clinical symptoms, resulting in different tumor outcomes [28]. In contrast to the BCLC stage, immune parameters are readily available in routine clinical practice and can be used to examine the balance between host inflammation and immune response based on variation trends for appropriate interventions. Notably, in our study, the AUROC was considerably better in the nomogram than the BCLC stage alone. Moreover, in our model, BCLC-B patients had worse prognosis than BCLC-C patients, which may be related to the following factors in the training cohort: (1) The number of BCLC-B patients with ≥ 4 tumors (15/24; 62.5%) was higher than that of BCLC-C patients (85/172; 49.4%); and (2) The number of BCLC-B patients with chronic HBV (23/24; 95.8%) was higher than that of BCLC-C patients (139/172; 80.8%). An increased number of tumors is associated with poor prognosis and poor prognosis in chronic hepatitis B-related liver cancer [29, 30]. Meanwhile, pathological spleen (including enlarged spleen) is an important risk factor for tumor recurrence and long-term survival [31]. In this study, enlarged spleen was also found to be an independent prognostic factor of uHCC patients. Therefore, the enlarged spleen index, which has been rarely investigated in studies but frequently occurs in clinical uHCC patients, was included, and it showed certain predictive ability in the line graph. Pathologically enlarged spleen is a significant factor influencing the poor prognosis of uHCC patients.

Despite these findings, there are several limitations to this study. First, this study was a retrospective analysis of a single institution, and our column map was verified internally. Second, our follow-up time failed to show a 5-year calibration curve. In addition, the mechanism of the systemic inflammatory response on tumor progression was not investigated in this study. Therefore, further research is needed to determine the detailed mechanism.

## Conclusions

In conclusion, post-SII and delta-NLR are independent prognostic factors of OS and may be promising indicators for clinical application. The nomogram based on post-SII and delta-NLR is very prominent in predicting OS in uHCC patients treated with IMRT. Further external validation of the nomogram is needed to jointly determine the potential patient benefit of IMRT as the primary treatment for uHCC.

## Supplementary Information

**Additional file 1.** Dynamic changes of complete blood counts and immune parameters pre-RT and post-RT

## Data Availability

The data underlying this article will be shared on reasonable request to the corresponding author.

## Abbreviations

HCC: Hepatocellular carcinoma
uHCC: Unresectable HCC
RT: Radiotherapy
NLR: Neutrophil-to-lymphocyte ratio
PLR: Platelet-to-lymphocyte ratio
SII: Systemic immune-inflammation index
IMRT: Intensity-modulated radiotherapy
CT: Computerized tomography
GTV: Gross tumor volume
PTV: Planned target volume
ROC: The receiver operating characteristic
AUROC: Area under the receiver operating characteristic curve
OS: Overall survival
BCLC: Barcelona clinic liver cancer
TACE: Transcatheter arterial chemoembolization
HR: Hazard ratio
