# Supplementary material for: Prognostic value of a nomogram based on peripheral blood immune parameters in unresectable hepatocellular carcinoma after intensity-modulated radiotherapy
Source: BMC Gastroenterol. 2022 Dec 9;22:510. doi: 10.1186/s12876-022-02596-0 (PMC9733385; doi:10.1186/s12876-022-02596-0)
Supplement: Supplementary file 1 — Additional file 1. Dynamic changes of complete blood counts and immune parameters pre-RT and post-RT [file 12876_2022_2596_MOESM1_ESM.docx]

Additional file 1 Dynamic changes of complete blood counts and immune parameters pre-RT and post-RT

| Parameter | Pre-RT | Post-RT | P value |
| --- | --- | --- | --- |
| Platelet count | 184.44 ±85.20 | 135.20 ±58.66 | <0.001 |
| Lymphocyte count | 4.53 ±4.74 | 3.44 ±3.90 | <0.001 |
| Neutrophil count | 1.91 ±10.52 | 1.14 ±4.30 | 0.298 |
| PLR | 168.28 ±150.25 | 353.96 ±450.60 | <0.001 |
| NLR | 4.52 ±6.05 | 9.26 ±13.77 | <0.001 |
| SII | 849.16 ±1323.86 | 1261.09 ±2206.24 | <0.001 |

Data are mean ± standard deviation.

PLR= platelet count/lymphocyte count; NLR=neutrophil count/lymphocyte count; SII = platelet count× neutrophil count/lymphocyte count. Pre-RT, before radiotherapy; Post-RT, 1 month after radiotherapy.
